# Coping with loneliness and stigma associated with HIV in a resource-limited setting, making a case for mental health interventions; a sequential mixed methods study

**DOI:** 10.1186/s12888-023-04643-w

**Published:** 2023-03-14

**Authors:** Jerry Paul Ninnoni, Sampson Opoku Agyemang, Lydia Bennin, Elizabeth Agyare, Leveana Gyimah, Kafui Senya, Nyonuku Akosua Baddoo, Francis Annor, Dorcas Obiri-Yeboah

**Affiliations:** 1 Department of Mental Health, School of Nursing and Midwifery, University of Cape Coast, Cape Coast, Ghana; 2 Public Health Unit, Cape Coast Teaching Hospital, Cape Coast, Ghana; 3 Communicable and Non-Communicable Diseases cluster, World Health Organisation Country Office, Accra, Ghana; 4 National AIDS/STIs Control Programme, Accra, Ghana; 5 Department of Community Health, the University of Ghana Medical School, Accra, Ghana; 6 Direcctorate of Research, Innovation and Consultancy, University of Cape Coast, Cape Coast, Ghana; 7 Department of Microbiology and Immunology, School of Medical Sciences, University of Cape Coast, Cape Coast, Ghana; 8 Department of Mental Health, School of Nursing and Midwifery, College of Health and Allied Sciences, University of Cape Coast, Cape Coast, Ghana

**Keywords:** Stigma, Loneliness, Coping strategies, HIV/AIDS, Mental health, Ghana

## Abstract

**Background:**

Challenges such as stigma and loneliness may increase vulnerability to Human Immunodeficiency Virus (HIV) infection and negatively affect the quality of life of people living with HIV (PLHIV) despite the massive investment in access to antiretroviral therapy. This study aims to determine the level of loneliness and stigma and explore the coping resources employed by PLHIV in a resource-constrained setting.

**Methods:**

This was a sequential mixed methods study conducted at the Cape Coast Teaching Hospital (CCTH) in Ghana between May and December 2021. A total of 395 adults were selected using a simple random sampling technique. HIV Stigma Scale and UCLA Loneliness Scale were used to collect quantitative data. A purposive sampling technique was applied to recruit 18 participants to saturation using a semi-structured interview guide. SPSS version 21 was used for the statistical analysis of the quantitative data. HIV-related loneliness and stigma levels were estimated, and bivariate and multivariable logistic regression were used to evaluate associated factors using a statistical significance of p-value (p < .05). In general, the thematic analysis approach by Braun and Clark was employed to analyse the qualitative data. Findings were then triangulated.

**Results:**

The mean age was 46.79 years (*±* 12.53), 75.4% of the participants were female, with a prevalence of stigma of 99.0% (95%CI = 97.4–99.7) and loneliness of 30.1% (95%CI = 25.6–34.9). Tertiary-level education and instrumental support were associated with lower levels of loneliness. In contrast, comorbidity, personalised stigma, negative self-image, and self-blame were positively related to loneliness. Thematic analyses of the qualitative data produced a range of themes that showed that people living with HIV rely on personal resources, social support networks, and behaviour modification strategies to manage their condition. In particular, some of these strategies include; religiosity and spirituality, family and friends, medication and professional support systems.

**Conclusion:**

The results suggest that PLHIV in the developing world face enormous challenges, socially, psychologically and financially. Although there have been global efforts to make HIV services accessible, the findings suggest a need for integrating mental health services contextually to reduce loneliness and HIV-related stigma to improve quality of life.

## Background

Human Immunodeficiency Virus (HIV) is one of the world’s highly stigmatised conditions, and it is estimated that 38 million people across the globe are living with it. About five million people in Western and Central Africa live with HIV [1]. For example, in Ghana, it is estimated that there are about 342,307 (122,321 males and 219,986 females) people living with HIV (PLHIV), with a prevalence rate of about 2% [2].

HIV stigma and loneliness remain global health problems. Challenges such as stigma and loneliness may increase vulnerability to HIV infection and negatively affect the quality of life of PLHIV despite the massive investment in access to antiretroviral therapy [1, 3–6]. Previous studies have reported that sources of stigma toward PLHIV comprise families (parents, siblings and relatives), communities and healthcare settings [4, 5, 7].

Goffman explained stigma as an attribute that links a person to an undesirable stereotype, leading other people to reduce the bearer from a whole to a tainted one [8]. Stigma is perceived as an undesirable trait that tremendously harms the reputation of an individual and abridges in our minds from a complete and usual personality to a stained, less valuable one [8]. Stigmatisation is a social process occurring in the context of power, where an individual’s differentness or attribute is considered unfavourable and linked to negative stereotypes [9]. The manifestations of stigma are socially constructed and context-specific; possessing such an attribute generally results in a loss of status, recognition, devaluation and discrimination leading to unequal outcomes for the stigmatised individual or population [8, 10, 11]. Further claims suggest that stigma is an ideology that identifies and links biological disease to a negatively defined behaviour [12].

Moreso, Goffman [8] outlined the distinction between “discredited” and “discreditable” identities. Discredited individuals may have a known or visible attribute, requiring them to devise coping mechanisms to manage the resulting prejudice and discrimination, and this is termed as “enacted stigma”. On the contrary, conditions which can be hidden from the public eye create discreditable identities, where the main focus is managing and concealing information to be accepted and “pass” as “normal” to avoid becoming discredited and experiencing the expected resultant stigma, which can also be referred to as “anticipated stigma” [8, 13]. Central to Goffman’s [8] notion of stigma is the issue of relationships. Goffman [8] posited that stigma exists because society labels an individual or a group as deviant. The labelling happens in many diverse settings, including mental health, COVID-19 and HIV [14]. In addition, the fear of contracting HIV through physical contact with PLHIV and the lack of knowledge about how the infection is transmitted has been reported to be the main drivers of stigma and discrimination [7, 15, 16].

Stigma and discrimination affect PLHIV in diverse ways and are considered barriers to quality health care [4, 17]. In addition, the persistent stigmatisation and discrimination toward PLHIV are associated with psychological distress such as stress, depression, loneliness and social isolation [4, 18, 19].

On the other hand, loneliness is an unpleasant experience when an individual’s social network of relationships feels significantly deficient [20]. It is a distressing discrepancy between preferred and actual levels of social interaction [21, 22]. Loneliness is an aversive emotional experience that arises from a perceived deficiency in the quantity or quality of social relationships [20].

Numerous theories have been forwarded to further the understanding of loneliness. The interactionists view loneliness as multidimensional, suggesting that there are different types of loneliness, including emotional and social loneliness [23]. Loneliness is caused by being alone and without the needed relationship or set of relationships. In many instances, it is a response to the absence of provision of a close, indeed intimate, attachment. It also may be a response to the lack of a meaningful friendship, collegial relationship, or other linkages to a coherent community [24]. The psychodynamic understanding of loneliness is reflected in the infant’s attachment to the mother. Through attachment, children experience emotional bonds, how to connect with others, and feelings of loneliness when significant others lack [25].

Furthermore, the existential perspectives also differentiate between different types of loneliness, with the main one being existential, suggesting that loneliness is normal and part of human existence but can also result from anxiety and, thus, become pathological [26]. However, empirical research led to two main theoretical approaches directly related to the conceptual definition of loneliness. These include the social and cognitive needs approaches [27, 28]. The social needs approach views loneliness as resulting from a breakdown of social conditions in childhood [29]; however, the cognitive approach claims that feelings of loneliness are due to individuals’ reactions to social situations—loneliness results from changes in social relationships or the desired or expected social relationship [30].

A survey found that about one-third of older adults were lonely [31]. These estimates are concerning because loneliness poses a significant risk for morbidity and mortality and is linked to poor outcomes [32, 33]. In addition, adults with HIV often face increased and complex vulnerability regarding physical and psychosocial needs; this may exacerbate loneliness [34]. Adults with HIV tend to live alone and have limited and inadequate social networks compared to their younger counterparts.

The incidence of loneliness is high among people living with chronic illnesses such as HIV compared to the general population due to stigma and discrimination [35]. Loneliness is an essential aspect of quality of life, and its’ consequences on health should not be underestimated [36]. On the other hand, chronic health conditions such as HIV, cancer, hypertension and mental illness can also serve as risk factors for the onset and persistence of loneliness [37–39].

Although other studies have investigated HIV-related stigma, most of these studies have broadly focused on the effects of stigma on the individual, leaving a gap in knowledge regarding the association between stigma and loneliness. This article presents findings from a project on mental health assessment for PLHIV in a Ghanaian setting [40]. This sub-study aims to determine the level of loneliness and stigma and explore the coping resources employed by PLHIV seeking care in Cape Coast Teaching Hospital and thus contribute to informing appropriate mental health-related Differentiated Service Delivery interventions to improve the quality of life of PLHIV.

## Methods

### Study design and study area

This is an explanatory sequential mixed methods study conducted at the Cape Coast Teaching Hospital (CCTH) in Ghana between May and December 2021. CCTH is the largest hospital and has the highest number of clients at the Antiretroviral Therapy (ART) clinic in the Central Region of Ghana. The hospital has a bed capacity of 400, and it is affiliated with the School of Medical Sciences and the School of Nursing and Midwifery at the University of Cape Coast, Ghana. Initially, the quantitative data was collected using a structured self-report questionnaire, and its findings informed the qualitative arm. The conclusions of the quantitative study informed the design of the interview guide. Subsequently, the qualitative component was conducted to triangulate the quantitative data and obtain a comprehensive picture of how PLHIV cope with loneliness and stigma.

### Population and sampling procedure

The population for this study included all adults (≥ 18 years) living with HIV/AIDS for at least 12 months who received care from the CCTH. Details of the recruitment process and sample size calculation for the quantitative aspect are as described by Opoku Agyemang et al. [40].

In brief, the sample size was predetermined statistically with a 95% confidence interval of 363. However, the sample size was increased by 10% to make provisions for incomplete data. Finally, a simple random sampling technique was applied to obtain the required participants for the quantitative arm.

Participants in the quantitative arm were not included in the qualitative study. Instead, a purposive sampling technique was used to recruit 18 participants for the study until data saturation. The inclusion criteria were; adults ≥ 18 years with a confirmed HIV/AIDS status for at least a year and receiving care at the Treatment Centre, as discussed in the quantitative arm. These individuals were approached during their routine clinic visits and provided detailed information regarding the study, including ethics, confidentiality and informed consent. Eligible participants who provided written informed consent to the study were allowed to decide on the interview place, date and time. Two consent forms were signed, one for the researcher’s records and the other for the participant. All interviews lasted for an average of 35 min. Saturation was reached by the 15th interview but confirmed with 3 additional interviews, totalling 18 participants.

### Measures

Data was collected using a self-report anonymous questionnaire and semi-structured interview guides for quantitative and qualitative data, respectively. The questionnaire had three sections. The first section focused on the socio-demographic characteristics of participants. The second section comprised the HIV Stigma Scale. The HIV Stigma Scale used in this study is the 10-item short version of the 40-item instrument designed to measure subjective perceptions of stigma by persons living with HIV/AIDS [41]. The scale consists of four subscales: personalised stigma, disclosure concerns, concerns with public attitudes, and negative self-image. Personalised stigma and negative self-image were measured with three items each, whereas disclosure and worries about public attitudes were measured with two items each. All the items were rated on a four-point Likert-type scale ranging from “disagree”-1 to “strongly agree”- 4. The responses from participants were summed to calculate the score for the subscales, with higher scores reflecting a high level of HIV-related stigma [42]. Sample items on the scale include “I have been hurt by how people reacted to learning I have HIV” (personalised stigma); “I worry that people who know I have HIV will tell others” (disclosure); “Most people with HIV are rejected when others find out” (public attitudes); and “Having HIV makes me feel unclean” (negative self-image). The Cronbach’s alpha for this scale in this study is 0.67. The cut-off scores for the composite HIV stigma scale include 0–15 (no stigma), 16–24 (mild stigma), 25–35 (moderate stigma), and above 35 (severe stigma).

The final section comprised UCLA Loneliness Scale [43]. UCLA Loneliness Scale is a 20-item scale designed to measure one’s subjective feelings of loneliness and social isolation. Participants rate each item as either O (“I often feel this way”)- 3, S (“I sometimes feel this way”)- 2, R (“I rarely feel this way”)- 1, N (“I never feel this way”)- 0 [44]. The scale comprises 10 positively worded and 10 negatively worded items. The scoring is based on a four-point scale with positive items reversed. High scores indicate a great expression of loneliness. The cut-off point includes: 20–34 [low loneliness], 35–49 [moderate loneliness], 50–64 [moderately high loneliness], and 65–80 [increased loneliness] [45]. Some example items are “How often do you feel that you lack companionship?”, “How often do you feel there is no one you can turn to?” “How often do you feel you are no longer close to anyone?” “How often do you feel isolated from others?” The scale had a coefficient alpha of 0.74 in this study.

### Data collection procedure

Seven research assistants with at least a bachelor’s degree were recruited to assist with the quantitative data collection. In addition, they were trained on the type of information and the ethical principles to uphold during the data collection. The questionnaires were administered privately to each participant by a trained research assistant in an office at the antiretroviral therapy clinic. It took approximately 20 to 30 min for each participant to complete the questionnaire. Participants who were not literate were assisted in completing the questionnaire. Three hundred and ninety-five (395) questionnaires were returned with complete data, yielding a response rate of 99%.

In the qualitative arm, in-depth face-to-face interviews were conducted using a semi-structured interview guide. Before the interview, all participants were allowed to choose pseudo names. The interviews were conducted at an assigned office at the antiretroviral therapy clinic of Cape Coast Teaching Hospital. The time for each discussion was negotiated with the participant. Each interview lasted approximately 35 to 40 min and audiotaped, and two facilitators from the research team conducted it. Interview questions explored loneliness and stigma experienced by PLHIV and any coping strategies they adopted to overcome these challenges. Interviews and analysis continued until data saturation was confirmed by conducting three additional interviews.

All data collection processes were conducted in the participant’s preferred language, either in English or the local language (Fante). In addition, each researcher kept a field journal in which personal reflections and biases were recorded.

### Data analysis

SPSS version 21 was used for the statistical analysis of the quantitative data. Frequencies and percentages were used to estimate the prevalence of HIV-related loneliness and stigma. Bivariate and multivariable logistic regression were used to evaluate socio-demographic factors associated with HIV-related loneliness and stigma. Statistical significance was based on the p-value (p < .05) and associated 95% confidence interval (95% CI).

In the qualitative arm of this study, each interview was transcribed verbatim into English by the interviewer fluent in both English and the local langue (Fante) and then checked for consistency and accuracy. Then, transcribed interviews were coded, after which themes and sub-themes were generated. Next, each team member independently read the verbatim transcript to identify patterns. Afterwards, the group met to confirm the expected theme participants expressed. The themes were then categorised according to the objectives of the study. Finally, results are presented using quotations from participants to validate the phenomena.

In general, the thematic analysis approach by Braun and Clark was employed to analyse the data [46]. The six phases that characterised this framework include;

*Familiarisation with the data* refers to reading and rereading the transcript to immerse yourself in the data.

#### Generating initial codes

codes are identified through systematic analysis of the data.

*Searching for themes* is constructed by reviewing the codes to identify areas of similarity and overlap between codes about the research question.

#### Reviewing potential themes

to check the data quality, the developing pieces are evaluated about the coded data and the entire data.

#### Defining and naming themes

what is specific and unique about each theme is clearly stated.

Producing the report: data collection and analysis are done concurrently. Writing and analysis are interwoven.

### Ensuring trustworthiness

Trustworthiness was ensured by checking the findings to ensure the transcriptions were accurate and assessing the consistency of themes by different research team members. Two team members read the transcripts and assigned codes to various categories of data. All codes identified had significant implications for the research question. The participants validated the findings through member checking [47]. The researchers kept an audit trail of all activities during the study.

### Ethics approval

This study was performed following the Helsinki Declaration, and the Ethics Review Committee of Cape Coast Teaching Hospital, Ghana, approved it (reference: CCTHERC/EC/2021/028). The purpose of the study, anonymity, voluntary participation and confidentiality of the information were explained to participants to seek their written informed consent since only adults were involved. Participants who formal education, the information was translated into the local language (Fante) and they then thumbprint the informed consent form in place of the signature. Participants were made aware that they could withdraw from the study. All COVID-19 protocols, such as; handwashing, social distancing and wearing of facemasks, were observed during the study.

## Results

### Demographic data of participants

The socio-demographic and other characteristics of the 395 participants for the quantitative arm are described extensively by Opoku Agyemang et al. [40]. The mean age was 46.79 years (*±* 12.53), and 75.4% of the participants were female ( see Table 1 below).

**Table 1:** Socio-demographic and clinical characteristics of participants (N = 395)

Characteristic	Category	n(% or SD)
Age (years)		
	Mean	46.7 (12.53)
	20–39	122 (30.8)
	40–59	206 (52.2)
	60–80	67 (17.0)
Gender		
	Male	97 (24.6)
	Female	298 (75.4)
Marital status		
	Currently with a regular partner	151 (38.2)
	Currently without a regular partner	244 (61.8)
Religion		
	Christian	355 (89.9)
	Islam	31 (7.8)
	Traditionalist	3 (0.8)
	No religion	6 (1.5)
Employment status		
	Employed by other	12 (3.0)
	Employed by self	307 (77.7)
	Unemployed	76 (19.2)
Educational level		
	Tertiary	38 (9.6)
	Non – tertiary	246 (62.3)
	No basic education	111 (28.1)

### Prevalence estimates of loneliness and stigma

The prevalence rate for HIV stigma was estimated based on the number of participants that reported mild to severe stigma. In contrast, the prevalence rate for loneliness was estimated based on the number of participants that reported moderately high to high degrees of loneliness. As shown in Table 2, the results indicate an extremely high prevalence of stigma (99.0%; 95% CI = 97.4–99.7) among participants in the study. On the other hand, loneliness (30.1%; 95% CI = 25.6–34.9) was moderate among the study participants. However, there were no noticeable differences between males and females in the prevalence rates of stigma and loneliness.

**Table 2:** Prevalence rates for loneliness and stigma by gender

		Variables				
	Stigma			Loneliness		
Characteristics	*n* (%)		95% CI	*n* (%)	95% CI	
Overall	391(99.0)		97.4–99.7	119 (30.1%)	25.6–34.9	
Male	95 (97.9)		92.8–99.8	30 (30.9)	21.93–41.1	
Female	296 (99.3)		97.6–99.9	89 (29.9)	24.7–35.4	

#### Bivariate associations between loneliness, stigma and correlates

Table 3 presents means and standard deviations for variables in the study and their bivariate correlations with loneliness. Dummy codes were created for level education with “no education” as the reference category, whereas the other categorical demographic variables were dichotomous. Among the demographic variables, comorbidity and education were significantly associated with loneliness. Specifically, participants who had other health conditions were more likely to express loneliness. Likewise, participants with a tertiary level of education were less likely to express loneliness than those without education. Personalised stigma, negative self-image, and self-blame had significant positive correlations with loneliness, whereas emotional support, instrumental support, and acceptance had significant negative correlations with loneliness.

**Table 3:** Descriptive statistics and the bivariate association between loneliness and correlates

Variable	Mean	*SD*	Correlation
Age	46.79	12.53	− 0.09
Gender	1.75	0.43	0.02
Comorbidity	1.83	0.37	0.13^*^
Tertiary education ^*a*^	0.10	0.30	–0.15^**^
Secondary education ^*a*^	0.16	0.37	–0.03
Basic education ^*a*^	0.46	0.50	–0.01
Employment status	1.19	0.39	0.06
Marital status	1.62	0.49	0.12^*^
Religious affiliation	1.10	0.30	0.07
Personalized stigma	7.07	1.96	0.32^***^
Negative self-image	6.49	2.07	0.25^***^
Public attitude	5.54	1.88	0.04
Disclosure	6.14	1.54	0.05
Active coping	5.69	1.83	− 0.02
Denial	4.19	1.91	–0.02
Substance use	3.02	1.65	0.10
Emotional support	5.42	1.90	–0.20^***^
Instrumental support	5.42	1.88	–0.21^***^
Behavioural disengagement	4.01	1.73	0.15^**^
Venting	4.71	1.81	–0.04
Positive reframing	5.44	1.64	–0.07
Planning	5.38	1.82	0.02
Humour	3.36	1.81	0.08
Acceptance	6.58	1.48	–0.13^*^
Self-blame	4.51	1.96	0.21^***^
Self-distraction	6.17	1.89	0.01
Religious coping	6.52	1.74	–0.04
Loneliness	45.09	9.15	

*Notes*. ***a*** reference category = no education; * *p* < .05; ** *p* < .01; *** *p* < .001

#### Multivariate associations between loneliness and correlates

Table 4 presents results for the sequential multiple regression analyses on correlates of loneliness. In Step 1, the demographic variables accounted for 5% of the variance in loneliness. In Step 2, stigma accounted for an additional 13% of the variance in loneliness. In Step 3, they were coping accounted for an extra 8% of the variance in loneliness. The final regression model was statistically significant (F (12, 382) = 11.40, p < .001; R^2^ = 0.26), and the variables accounted for 26% of the variance in loneliness. The final regression model showed that a tertiary education level (β = –0.16, p < .01) was associated with a lower likelihood of loneliness than no education. Likewise, the presence of a comorbid condition was associated with a higher chance of loneliness (β = 0.13, p < .01. Personalized stigma (β = 0.24, p < .001) and negative self-image (β = 0.16, p < .001) had significant positive relationships with loneliness. Among the coping strategies, instrumental support (β = –0.20, p < .001) and self-blame (β = 0.12, p < .01) had significant negative relationships with loneliness.

**Table 4:** Summary of sequential regression analysis on correlates of loneliness

	*Step 1*			*Step 2*			*Step 3*		
Variable	*b*	*S.E.*	*β*	*b*	*S.E.*	*β*	*b*	*S.E.*	*β*
(Constant)	38.93	2.95		24.23	3.33		30.40	4.03	
Comorbidity	2.67	1.22	0.11^*^	3.09	1.14	0.13^**^	3.11	1.10	0.13^**^
Tertiary education	–5.30	1.70	–0.17^**^	–4.98	1.59	–0.16^**^	–4.84	1.54	–0.16^**^
Secondary education	–2.21	1.41	–0.09	–2.16	1.32	–0.09	–2.48	1.27	–0.10
Basic education	–1.72	1.09	–0.09	–1.13	1.02	–0.06	–1.07	0.98	–0.06
Marital status	1.81	0.93	0.10	1.66	0.87	0.09	1.61	0.83	0.09
Personalized stigma				1.22	0.23	0.26^***^	1.13	0.22	0.24^***^
Negative self-image				0.81	0.21	0.18^***^	0.72	0.21	0.16^***^
Emotional support							–0.07	0.27	–0.02
Instrumental support							–0.97	0.27	–0.20^***^
Disengagement							0.42	0.26	0.08
Acceptance							–0.54	0.30	–0.09
Self-blame							0.57	0.22	0.12^**^
*R* ^2^			0.05			0.18			0.26
Δ*R*^2^						0.13			0.08
Model *F*			4.42^**^			12.34^***^			11.40^***^

*Notes*. ***a*** reference category = no education; * *p* < .05; ** *p* < .01; *** *p* < .001

## Qualitative ARM

### Demographic data of interview participants

As shown in Table 5, most respondents were women (n = 13), and few were married (n = 5). In addition, the majority of them were 40 years older (n = 14), and most had lived with HIV diagnosis for five years or more (n = 14).

**Table 5:** Demographic Data of Interview Participants, N = 18

No.	Pseudonym	Gender	Age (yrs)	Marital status	Religion	Employment Status	Education level	Years of HIV diagnosis
1.	Adwoa	Female	48	Divorced	Christian	Unemployed	Primary school	4
2.	Kwame	Male	45	Divorced	Christian	Unemployed	SHS	6
3.	Joyce	Female	40	Divorced	Christian	Employed	Middle school	9
4.	Ama	Female	67	Widowed	Christian	Unemployed	Middle school	5
5.	Rita	Female	31	Cohabiting	Christian	Employed	SHS	7
6.	Serwaa	Female	42	Cohabiting	Muslim	Employed	Middle school	10
7.	Yaa	Female	38	Divorced	Christian	Employed	Vocational	8
8.	Lizzy	Female	46	Married	Christian	Employed	None	12
9.	Daniel	Male	72	Married	Christian	Unemployed	Middle school	5
10.	Emmanuel	Male	69	Married	Christian	Employed	Middle school	7
11.	Elorm	Female	55	Widowed	Christian	Employed	None	6
12.	Kofi	Male	52	Divorced	Christian	Employed	Middle school	3
13.	Sandra	Female	47	Single	Christian	Employed	None	13
14.	Akosua	Female	40	Married	Christian	Employed	SHS	4
15.	Esther	Female	56	Divorced	Christian	Unemployed	Middle school	9
16.	Joseph	Male	70	Divorced	Christian	Employed	Middle school	11
17.	Millicent	Female	38	Married	Christian	Employed	JHS	5
18.	Esi	Female	33	Single	Christian	Employed	Tertiary	3

The analysis produced two main themes; psychological experience and available coping resources. Subthemes of the psychological experience include stigma and loneliness.

**Fig. 1:**
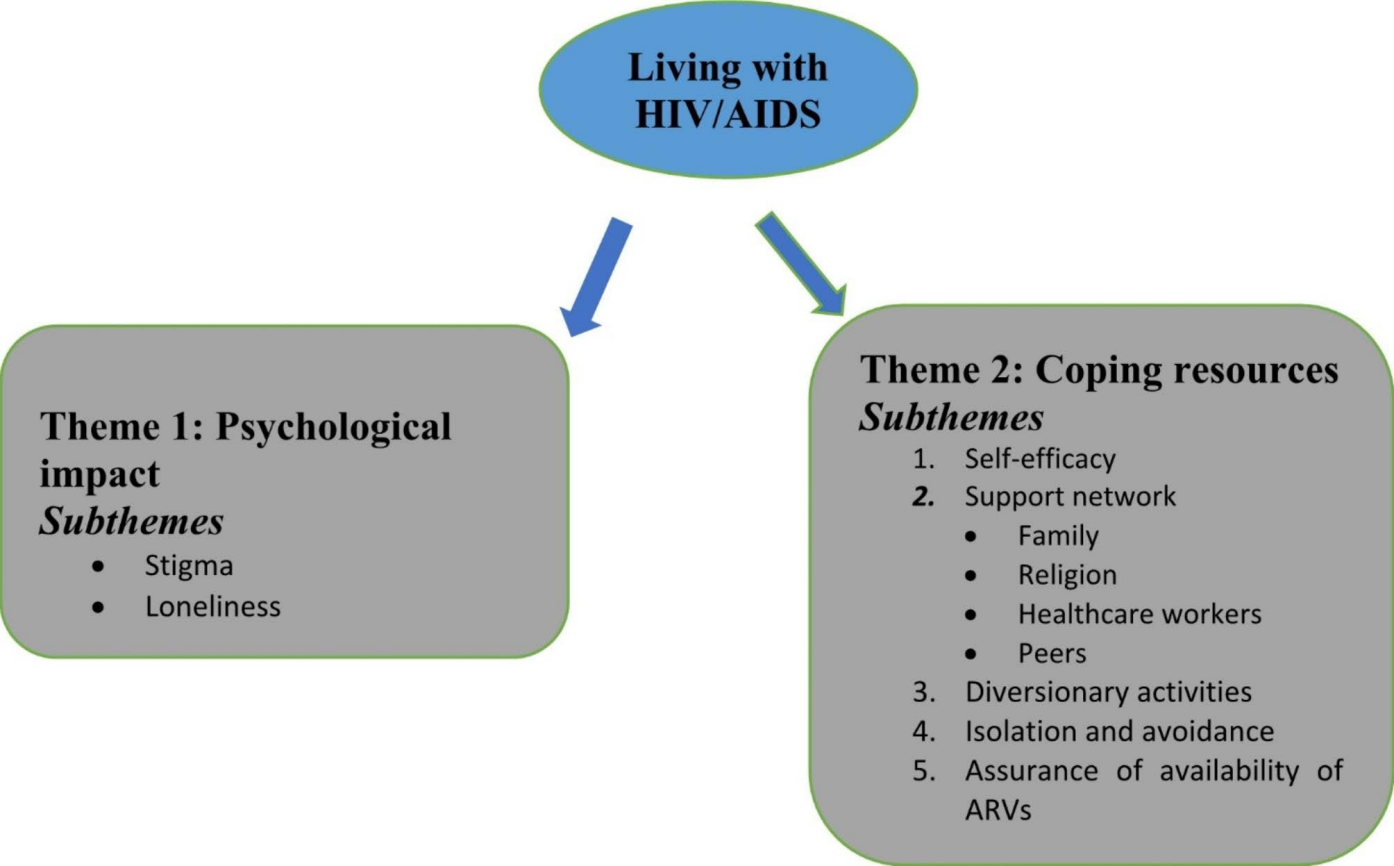
Theme and Sub-themes

In addition, participants described a range of available resources and strategies they adopt to cope with the disease. These include support networks, medication adherence, isolation/avoidance, self-efficacy and diversionary activities, as shown in Fig. 1 below.

### Psychological experiences

The psychological impact resulting from HIV/AIDS is primarily related to HIV-related stigma and loneliness and reflects the findings from the study’s quantitative arm. Therefore, this section will present the qualitative results and integrate them into the discussion section.

#### HIV-related Stigma

Participants reported various forms of HIV-related stigma. These included self or internalised stigmata, public stigma, and, to a lesser extent, stigma from professionals. Stigma leads to poor health-seeking behaviours, disempowerment, reduced self-efficacy and decreased quality of life.

### Self-stigma

Respondents also reported self-stigma. Several respondents expressed that living with HIV made them feel worthless. This was described in various forms in the words of respondents as follows;

*“When I cough, I normally don’t share cups with anybody. I have my spoon; I used my plate; I use my everything since I have the disease, so that’s it,”* Participant Adjoa.

She further observed that discrimination is inevitable for one to have the disease.

*“No matter what it is, there will be some discrimination sometimes; Mummy, for example, is the one I spend much time with, but at times, she tells me to be very careful with her. As you know, she gets angry sometimes”. My brother, when he comes to visit, he doesn’t sleep overnight; he tells us he’s busy, but I think it’s because of me, but I don’t bother, not that he goes about telling people, but always that’s what he says, he’s busy”* Participant Adjoa.

For fear of public ridicule and discrimination, she doesn’t disclose her HIV status apart from her family.

*“As I said before, I have not told anyone apart from my mother and siblings and my two uncles. Nobody knows; even my friends don’t know. Because I have this thing, I am not too public, although I used to be. I stop outings, even the beach. I quit going to the beach because of that. I have to be very careful,”* Participant Adjoa.

*“Yes, because it makes me feel unclean; how people talk and behave towards you when they hear you have this disease is even embarrassing. People will hear about it and broadcast me; I feel shy and sad too,”* P*articipant Ama.*

*“I can’t tell anyone and am unwilling to do that because this disease is embarrassing”.* Participant Lizzy

### Public/social stigma

Participants narrated the negative attitudes towards them by the general public, often based on false beliefs, fear and prejudices. The participants indicated they were stigmatised since they were diagnosed with HIV/AIDS. Some participants said even their family members stigmatised them.

*“In this life, there are people that whatever they hear, they spread it everywhere, so that’s why I don’t want anybody to know apart from my family”.* Participant Adjoa

.

*“My children have even suggested I go to a private hospital for treatment so that people won’t get to know, but because of diabetes or maybe when it worsens, they will still refer me to this place, so I decided to be here. I will feel embarrassed if people get to know about it*”. Participant Ama

*“ People think that if you involve yourself in prostitution, that is how you get it, so I was wondering what people will think about me when they know about it”.* Participant Serwaa

*“They may even avoid you; you can’t buy food or something, and they won’t sell to you. They keep pointing fingers at you anytime you pass by, and because of the disease, some older adults wouldn’t come here. So the stigma and discrimination will not even make people come here”.* Participant Rita.

Another participant also reported this experience:

*“Sometimes too, when I go to my customers for the food stuffs I sell, they refuse to sell to me because they thought I can’t pay them early or may not pay them at all because of how lean I have become”*, Participant Serwaa.

Participants are somehow paranoid about what people say regarding their condition:

*“Yes. Because some people can’t keep their mouths shut, they will discuss it wherever they go and disgrace you. In the beginning, people said all sorts of things about the disease, so when they know you have it, they make it look like you did something bad that has landed you in that trouble,”* Participant Serwaa.

*“There is no problem coming here, but I get worried I may meet someone I know who does not have the disease and will broadcast me. So if I see such people, I hide my face from being seen until the person leaves,”* Participant Akosua.

However, a participant experienced professional stigma:

*“When I went to the hospital and found out I have the disease, they asked me to go and treat it, so I lost my job; yes, I had to accept it even though they relate well with me”*, Participant Adjoa.

### Loneliness

The participants expressed how HIV/AIDS negatively affected their interpersonal relationships. The condition made it difficult for them to associate well with others, including finding a life partner. The respondents demonstrated this feat in the following narratives;

*“Me, I don’t get help from anyone. Even in my family, most Muslims are only used to their Muslim brothers and sister. If you go to them, they only listen to your message and tell you it will be well, but they don’t help you in any way, so the little I earn is what I manage with my children”.* Participant Adjoa.

A participant narrated the challenges in finding a partner:

*“We have challenges with relationships, so if they can get us contacts of people who also have the condition, it may help us because if you marry someone who does not have the condition, sometimes, you can’t take your medicines when he is around.”* Participant Millicent.

*“I would wish that time for consultation, especially among the youth, if any man who also has the disease and needs a wife, they could link us because if you go for someone who does not have the disease, they will leave you if they find out… and also they can help get work to do to support us”* Participant Yaa.

Some participants reported a lack of friendship leading to loneliness:

*“I don’t have tight friends I go to or sometimes, maybe we meet at the market in place morning, then I go to my house but to go to their house, I don’t do that. I don’t live with my family; I live outside Ghana, but when I come home, we are fine”.* Participant Elorm.

*“The reason is that the way they describe the disease makes people isolate themselves. They will also be pointing their fingers at you. This can get you thinking, so I better keep it to myself”.* Participant Akosua.

*“Yeah, there are several worries; for instance, since my husband died, I am the only person catering for my children. I can’t marry again, at least for the person to support me financially”.* Participant Elorm.

### Theme two: coping strategies

This theme characterises the various dimensions of support available to respondents to help them manage HIV/AIDS in the community. Under this theme, five [5] sub-themes emerged, including self-efficacy, support network (family, religion, healthcare workers and peers), diversional activities, isolation and avoidance, and treatment adherence.

### Self-efficacy

Some participants demonstrated self-sufficiency by stating that they were not bothered by the disease HIV/AIDS) because they were living in their own house. Expressly, one respondent stated;

“*I have no such problem; I live in my own house, so I don’t encounter those things that can make me uncomfortable. I am free, and I don’t think about it at all. I know it has happened already, so I don’t worry about it anymore,” said* Participant Emmanuel.

Some participants also opined that they were not in this alone since other people they perceived to be better off in society had also contracted the disease. To these participants, people are living with other conditions freely in the community, so their case was no exception. Thus, they had to accept their fate and use the appropriate mechanisms to deal with the disease (HIV/AIDS).

*“As I am saying, people have different kinds of sickness, but they are walking around, so if I have this disease, it doesn’t mean I am useless”*, Participant Sandra.

Another respondent indicated, *“… I have accepted the situation. …this is what has befallen me, and no matter what I do, I have gotten the disease, so I have to look for a way to protect myself and go through it, so I am not bothered. Some people have not yet accepted their condition, so they are roaming from one prayer camp to the other, but I have accepted my fate that I can’t do anything about it*,” Participant Esi.

### Support network

The second sub-theme identified was the support network. Four sub-themes were generated: family, religion, healthcare workers, and peers. Some participants revealed how they received financial support, encouragement, companionship, and inspiration from their family members, friends, healthcare workers, and religious organisations. Below are some experiences shared by some respondents.

### Family

For some participants, family members were a source of inspiration, encouragement, and financial support. In particular, the participants indicated that they gain financial support from siblings, in-laws, and their children. Additionally, some married participants said they enjoyed companionship from their spouses.

“…*people who know me encourage me, and I also make sure to take my drugs well”*, Participant Serwaa.

Another participant averred,

*“…one of my brothers is a medical doctor at Korle-Bu; sometimes he sends me money for other drugs and upkeep”*, Participant Yaa.

One male respondent also added,

*“…mmm, apart from my siblings, I don’t get help from anybody. They support me financially. At times, they buy things for me… Even my in-laws are all good to me,”* Participant Kwame.

Lastly, one male respondent added that he could cope because of the companionship he received from his wife. He said,

*“I was fortunate my wife didn’t have it yet; she supported me till this time*”, Participant Daniel.

The inspiration participants received from their families were expressed in the view of one participant as follows;

*“…my mother reminds me to take my drugs and encourages me to eat well. My other siblings who stay at a different place too, we communicate on the phone, and I visit them, we interact well,”* Participant Kwame.

### Religion

Some respondents relied on their beliefs and engagement with religious activities to cope and live meaningfully with HIV/AIDS. The respondents presented their views regarding God to portray how their faith is rooted in their religious background while living with the disease. The opinions expressed by respondents are evident in the following narratives;

*“The nurses made me understand that if I take my medicine well, I can live comfortably, but who kept me going is my reliance on God”*, Participant Lizzy.

*“First of all, I thank God because it wasn’t easy for me since I was diagnosed with the disease. It Is only God who has kept me going,”* Participant Kwame.

### Healthcare workers

Supportive healthcare workers and the healthcare system enhanced participants’ ability to cope with HIV/AIDs. Some participants stated that the encouragement the doctors and nurses gave them and the services provided at the clinic kept them going as reported by a participant;

*“…the nurses and the doctors are all good. Even when I default, they caution me and take good care of me. They are nice; when I compare how the nurses treated us when my mother felt sick in our hometown, I say the nurses here are good. Last time they suggested I transfer because I come from afar, but I refused because I like how they care for me here,”* Participant Kofi.

A female respondent added, *“The doctors and nurses have been very supportive. They treat me well anytime I come to the hospital, especially the elderly ones among them; she is always nice to me, even when she sees me in town. They are all good; even those who pick up our folders when we come to the clinic are nice to us. Sometimes when I cannot come for my medicines, they collect them and bring them to my house,”* Participant Joyce.

### Peers

Some respondents shared that friends and peers encouraged and supported them financially, enabling them to cope well with the disease burden.

*“…that my friend also encourages me. Sometimes when I don’t have money to come to the clinic, she even gives me money to come. She ensures I am doing better,”* Participant Rita.

*“…some of my friends also help me sometimes even though I have not told them anything”*, Participant Yaa.

### Diversionary activities

Another vital sub-theme emerged from the data related to respondents’ activities to redirect their attention and worries about living with HIV/AIDS. Some of these activities include music and watching movies. A female participant narrated;

*“…I listen to music a lot, which takes away my worries. I like watching movies too, so I don’t think about it,”* Participant Yaa.

Other respondents also focused on their work to keep them busy from worrying about their situation.

*Again, my work also engages me a lot and makes me busy. I am a teacher, so sometimes some students can behave to amuse you, which takes my attention from the disease,”* Participant Esi.

*“I focus on my work, I am always busy, so I don’t have time to be thinking about the disease”*, Participant Kofi.

### Isolation and avoidance

Another vital sub-theme from the data suggests that participants use isolation and avoidance to cope with the condition. They sometimes avoided working among people to escape gossip about their current status. One participant narrated;

*“…the way people make you feel when they know you have it (HIV) makes life difficult for you. At times, you don’t feel comfortable working among people,”* Participant Yaa.

*“…I have to avoid places where people are likely to gossip about me”*, Participant Lizzy.

### Assurance of availability of ARTs

The last sub-theme that emerged from the data was the availability of antiretroviral. This sub-theme concerned participants’ reliance on antiretroviral for survival and healthy living. The participants expressed this as follows;

*“I am encouraged by the fact that there is medicine to take to live healthily. In the past, if you get it (HIV), you die early, but I have lived with it for many years, and I am still healthy,”* Participant Elorm.

Another participant added:

*“I think the medicine because when you take them, you look ok. Also, the way I perceived the disease when I went for counselling and the teachings the nurses gave me made me understand that the disease is not as strange as people think”* Participant Kofi.

Another participant indicated, “I follow the teaching the doctors and nurses give *us. They counsel you well before treating you, so I listen to them and eat what they teach me.* Participant Esther.

## Discussions

The findings revealed a high prevalence of stigma among PLHIV, with an overall majority of 99%. Most study participants were females, consistent with previous studies [48, 49]. As captured in the study’s quantitative and qualitative arms, it was evident that stigma was a significant public health problem affecting PLHIV. HIV-related stigma remains high among people living with HIV in a resource-constraint setting like Ghana, with substantial implications for physical and mental health [50, 51]. The qualitative arm of the study, in particular, has revealed three forms of stigma; self-stigma or internalised stigma, public stigma and, to a lesser extent, professional stigma. It was further noted that PLHIV experience stigma from the day they are diagnosed with HIV, and their family members and peers are significant sources of HIV-related stigma. This is in agreement with previous studies, which indicate that PLHIVs face rejection from their families, workplace, school and members of the healthcare system [51, 52]. In addition, PLHIV expressed self-stigma, which affected their interpersonal relationship and made them feel worthless living with the condition.

Similar studies indicate that PLHIVs experienced internalised stigma, which affected their relationship negatively and increased their sense of loneliness [36, 50, 52, 53]. Stigma is an undesirable, discrediting attribute that an individual possesses, thus reducing the individual status in society [8]. It is a robust social process deeply rooted in social and cultural contexts [8]. Central to the themes of Goffman’s stigma theory is that society classifies people as ‘normals’ and ‘deviants. Individuals regarded as ‘deviants’ are stigmatised because they are said to be discounted and tainted and possess an undesired differentness from what the ‘normals’ anticipated [8, 54]. Society links a biological disease condition’s presence with a negatively defined behaviour and presentation.

Globally, HIV-related stigma remains high in the general population [55]. This may be related to a lack of education, ignorance, inaccurate information about HIV/AIDS, fear and moral judgement of the behaviours of those infected with HIV/AIDS [56]. In Africa, HIV is mainly associated with promiscuous heterosexual relationships. Attributing stigmatised medical condition such as HIV to a lack of personal responsibility tend to distance the ‘moral majority’ from risk [12, 57]. The study also showed a statistically significant relationship between stigma and education. Only a few of the study participants in this study had tertiary education. The results showed that individuals with higher education reported less stigma than those with lower educational qualifications. These findings are consistent with previous studies, suggesting increased knowledge is associated with decreasing stigma [58]. Although there was no statistical difference between gender and stigma in this study, females and higher education are reported as protective factors against stigma [48]. The consequences of HIV-related stigma include social rejection, social isolation, health inequalities, poor health outcomes and human rights abuses [59].

The study further revealed the occurrence of loneliness (30.1%) among PLHIV. Similar observations were made in the qualitative arm of this study, where participants reported loneliness influenced by stigma. Some reported experiences included; a lack of support from family and friends and a lack of partners. Other studies among people with chronic conditions also indicated the moderate prevalence of loneliness [36, 51]. They expressed that HIV/AIDS negatively affect their interpersonal relationships, especially in marriage. This makes it difficult for them to associate with others, including finding a life partner leading to loneliness, as reported in this study. [27, 28]. These findings reflect relevant theories of loneliness in the literature. The social needs approach views loneliness as resulting from a breakdown of social conditions [29], and the cognitive approach claims that feelings of loneliness are due to individuals’ reactions to social situations—loneliness results from changes in social relationships or the desired or expected social relationship [30]. PLHIVs find it challenging to settle with a life partner who will understand and cope with their current state [50]. Previous studies suggest that adults living with HIV/AIDS often face increased and complex vulnerability regarding physical and psychosocial needs, which may exacerbate loneliness [34]. The presence of discredited and discreditable identities in this study may suggest that to avoid discrimination, people may hide or avoid situations in which they may feel uncomfortable due to stigma and discrimination.

The current study found no significant differences between males and females in the prevalence rates of stigma and loneliness. This finding corroborates observations by Flickinger et al., where stigma had no significant association with age and gender in the PLHIV [53].

However, comorbidity and education were found to be significantly associated with loneliness. Specifically, PLHIV who had other health conditions were likelier to express loneliness. Also, PLHIV with tertiary education were less likely to express loneliness than those without formal education. This evidence suggests that formal education of PLHIV to a tertiary level may have increased their health literacy and formed a network of friends with a better understanding of HIV/AIDS infections, thus reducing stigma. On the other hand, HIV-related stigma may lead to shame and non-disclosure, leading to loneliness [34, 60]. Social marginalisation linked to HIV-related stigma and non-disclosure status is a common barrier to building social relationships [61].

Furthermore, the qualitative arm of the study, in particular, revealed a range of coping strategies as protective factors for living with HIV/AIDS. Goffman describes stigma and has a lot to say about its management. How does the stigmatised individual respond to situations? Goffman claims that, in some cases, it will be possible for the individual to attempt to correct what he sees as the basis for his failings. For example, when a physically disabled person undergoes plastic surgery, the blind person seeks eye treatment and illiterate remedial education [62]. Thus, this study goes beyond just documenting experiences and prevalence but identifies ways of overcoming loneliness and stigma through self-efficacy, diversional activities and social support networks, including religion and spirituality. In addition, concealment/avoidance of healthcare workers is a reported coping strategy for stigma. These findings are in congruence with Goffman’s propositions about ‘discredited’ and ‘discreditable’ identities and numerous previous studies, which report that help from family and friends positively impacts coping with HIV-related loneliness and stigma [63, 64]. A survey into coping strategies among people living with HIV/AIDS summarises coping strategies into four categories: compassion, social support, hiding HIV status and self-care [65]. This study’s participants reported concealment/hiding to cope with the stigma. It is claimed that status concealment is an information management strategy through which PLHIV maintain a discreditable identity and avoids becoming discredited [8]. In his seminal work, Goffman distinguishes between ‘discredited’ and ‘discreditable’ identities. Discredited individuals have evidence of visible attributes requiring them to devise coping mechanisms to manage the resulting prejudices and discrimination, which can also be termed as the ‘enacted’ stigma [8].

Conversely, conditions that cannot be hidden from the public create discredited identities. The main focus, in that case, is managing and concealing or disguising the condition to pass as ‘normal’ to avoid becoming discredited and experiencing the resultant stigma, also known as ‘anticipated’ stigma [64]. Because of their awareness of people’s reactions towards them in society, people in this study resort to concealing their identity to avoid prejudices and discrimination. These findings are consistent with a survey in China [65] but contradict other African studies. For example, Makoae and colleagues [66] conducted a study in five African countries and found that 21% of the participants were open about their diagnosis and served as spokespersons for others. These observations represent the two forms of resisting stigma [62]. First, members of a given stigmatised group themselves who act as representatives of the group before the audience of ‘normal’ and of the stigmatised and second, individuals who have achieved a very high degree of ‘normality’ such as families, healthcare providers and yet take it upon themselves talk against stigma [62]. A similar study found that about 81% of participants told at least one person within three months after diagnosis [67].

Affirming self-worth, support from family and reframing HIV status in a bright light have been noted to positively impact coping with loneliness and stigma associated with HIV [53]. In addition, self-acceptance of HIV status is reported to counter stigma and help individuals to overcome the fear and judgement of others and not internalise stigmatising attitudes and shame [57]. Also, the study revealed that PLHIV values financial support and encouragement from family. In particular, they gained financial support from siblings, in-laws, and their children, enabling them to meet their needs and cope with their condition. This finding is corroborated by another study which reported that social support significantly reduced loneliness and stigma among people living with chronic conditions [36, 50]. Hence support from spouse and family is essential in coping with a diagnosis of HIV-related loneliness and stigma.

Furthermore, the study findings revealed that PLHIVs who were self-sufficient and lived in their own house appeared to cope better with their condition than those who relied on others for basic needs. This finding is in congruence with a study that reported that PLHIV who are financially independent and engage in income-generating activities experience reduced feelings of self-stigma [50].

This study was conducted in a resource-limited setting where resources at individual and national levels are constrained, and people struggle to make a living. Although antiretroviral medications are provided freely, some people cannot afford to visit the clinic for medications as a result of financial constraints, and this can influence adherence and recovery. Furthermore, the African continent is highly religious, where people put faith above all things in moments of difficulty. Thus, religion and spirituality appeared as central sources of hope for coping with HIV/AIDS. Faith in God as a coping strategy reflects similar studies in the literature [68].

In addition, our findings revealed that doctors, nurses and a supportive healthcare system reduced the effects of loneliness and stigma on PLHIV. Encouragement from healthcare workers and quality service provided at the ART clinic served as coping mechanisms for PLHIV. PLHIV in ART experienced less stigma than those not in ART [36]. It is claimed that ART offers the potential to maintain good health and a hidden stigma [57]. ART was a means to prevent signs or symptoms of ill health from developing, which could expose one’s HIV status. Avoiding HIV visibility appeared more important than maintaining good health itself, with the fear of being identified being more pervasive than the fear of experiencing sickness [57]. This ability of ART to lessen physical manifestations of HIV has led to it being referred to as a technology of “invisibilisation”(69, providing social and economic opportunities and therefore providing PLHIV with a sense of value [69].

It is evident in this study that participants value institutional support from healthcare professionals and, in particular, doctors and nurses to improve their quality of life. In addition, establishing effective communication and person-centred therapeutic relationship with clients will promote treatment adherence and reduce stigma and related factors such as loneliness. This finding is reported in a similar study which emphasises that a relationship of trust between patients, doctors, nurses and other team members is one factor that positively influences adherence [68].

## Conclusion

HIV-related stigma in all its forms and loneliness remains a challenge and negatively impacts the lives of PLHIV. The qualitative and quantitative findings reveal that HIV-related stigma and loneliness remain pervasive and affect the quality of life. Therefore, there may be a need to relook at HIV-related psychosocial interventions regarding their effectiveness across socio-cultural settings. Also, it was evident from this study that internalised, or self-stigma, was commonly reported among PLHIV, and thus there is a need to target evidence-based, context-appropriate interventions. Also, some contextual coping strategies in terms of support networks, cultural beliefs and values may provide valuable channels of supporting and complimenting contemporary approaches for people with HIV/AIDS to improve their quality of life.

### Limitations

- This study is cross-sectional, but a follow-up with a longitudinal study may give a better picture of how participants experience stigma and loneliness over time.
- The participants were recruited in a hospital treatment centre where people attend for their ART. However, this approach may miss out on individuals unable to participate due to stigma and loneliness.
- Although consistent with the literature, especially in Africa, most of the participants are women and Christians, which may require further investigation to explore the experience of other demographic groups.

## Data Availability

All relevant data are included in this manuscript but can be available through a reasonable request to the corresponding author.

## List of abbreviations

PLHIV: People living with HIV
ART: Antiretroviral Therapy
SDG: Sustainable Development Goals
CCTH: Cape Coast Teaching Hospital
HIV: Human Immunodeficiency Virus

## References

[1] UNAIDS. Global HIV Statistics. Fact Sheet 2021. 2021;(June):1–3.

[2] Ghana AIDS (2019). Commission. Ghana’ s HIV fact sheet. Off Pres Ghana.

[3] Feyissa GT, Lockwood C, Woldie M, Munn Z (2019). Reducing HIV-related stigma and discrimination in healthcare settings: a systematic review of quantitative evidence. PLoS ONE.

[4] Tran BX, Ho RCM, Ho CSH, Latkin CA, Phan HT, Ha GH et al. Depression among patients with HIV/AIDS: Research development and effective interventions (gapresearch).Int J Environ Res Public Health. 2019;16(10). 10.3390/ijerph16101772 PMC6571985 31109139

[5] Jackson-Best F, Edwards N (2018). Stigma and intersectionality: a systematic review of systematic reviews across HIV/AIDS, mental illness, and physical disability. BMC Public Health.

[6] Lowther K, Selman L, Harding R, Higginson IJ. Experience of persistent psychological symptoms and perceived stigma among people with HIV on antiretroviral therapy (ART): a systematic review. Int J Nurs Stud. 2014 Aug;51(8):1171–89. 10.1016/j.ijnurstu.2014.01.015 24602830

[7] Halli SS, Khan CGH, Moses S, Blanchard J, Washington R, Shah I (2017). Family and community level stigma and discrimination among women living with HIV/AIDS in a high HIV prevalence district of India. J HIV/AIDS Soc Serv.

[8] Goffman E (1963). Stigma: notes on the management of Spoiled Identity.

[9] Link BG, Phelan JC. Stigma and its public health implications. Lancet. 2006 Feb;367(9509):528–9. 10.1016/S0140-6736(06)68184-1 16473129

[10] Gilbert L, Walker L (2010). My biggest fear was that people would reject me once they knew my status.”: Stigma as experienced by patients in an HIV/AIDS clinic in Johannesburg, South Africa. Heal Soc Care Community.

[11] Link BG, Phelan JC. Conceptualizing Stigma. Annu Rev Sociol. 2001 Aug;27(1):363–85.

[12] Deacon H, Stephney I, Prosalendis S (2005). Understanding HIV/AIDS Stigma: a theoretical and methodological analysis.

[13] Scambler G. Health-related stigma. Sociol Health Illn. 2009 Apr;31(3):441–55. 10.1111/j.1467-9566.2009.01161.x 19366430

[14] Ninnoni JPK, Addo MA. Recovery, stigma and COVID-19: a difficult puzzle.Br J Ment Heal Nurs. 2021 Jan2;10(1):1–3.

[15] Geter A, Herron AR, Sutton MY. HIV-Related Stigma by Healthcare Providers in the United States: a systematic review. AIDS Patient Care STDS. 2018 Oct;32(10):418–24. 10.1089/apc.2018.0114 PMC6410696 30277814

[16] Mahamboro DB, Fauk NK, Ward PR, Merry MS, Siri TA, Mwanri L. HIV Stigma and Moral Judgement: Qualitative Exploration of the Experiences of HIV Stigma and Discrimination among Married Men Living with HIV in Yogyakarta. Int J Environ Res Public Health 2020 Jan 19;17(2):636. 10.3390/ijerph17020636 PMC7013688 31963807

[17] Han S, Hu Y, Wang L, Pei Y, Zhu Z, Qi X (2021). Perceived discrimination and mental health symptoms among persons living with HIV in China: the mediating role of social isolation and loneliness. AIDS Care - Psychol Socio-Medical Asp AIDS/HIV.

[18] Rasoolinajad M, Abedinia N, Noorbala AA, Mohraz M, Badie BM, Hamad A et al. Relationship Among HIV-Related Stigma, Mental Health and Quality of life for HIV-Positive Patients in Tehran. AIDS Behav. 2018 Dec 2;22(12):3773–82. 10.1007/s10461-017-2023-z 29297112

[19] Ho S-S, Holloway A. The impact of HIV-related stigma on the lives of HIV-positive women: an integrated literature review. J Clin Nurs. 2016 Jan;25(1–2):8–19. 10.1111/jocn.12938 26234952

[20] Perlman D, Peplau LA, Peplau LA, Perlam D (1982). Perspectives on loneliness. Loneliness: a sourcebook of current Theroy, Research and Practice.

[21] Leigh-Hunt N, Bagguley D, Bash K, Turner V, Turnbull S, Valtorta N et al. An overview of systematic reviews on the public health consequences of social isolation and loneliness.Public Health. 2017Nov;152:157–71. 10.1016/j.puhe.2017.07.035 28915435

[22] Donovan NJ, Wu Q, Rentz DM, Sperling RA, Marshall GA, Glymour MM. Loneliness, depression and cognitive function in older U.S. adults.Int J Geriatr Psychiatry. 2017May;32(5):564–73. 10.1002/gps.4495 PMC5102822 27162047

[23] Sønderby LC, Wagoner B, Loneliness (2013). An Integrative Approach. J Integr Soc Sci.

[24] Weiss RS (1989). Reflections on the Present State of Loneliness Research. Og. IHM, editor.

[25] Sullivan HS. The interpersonal theory of psychiatry. An Introduction to Theories of Personality. 7th ed.Psychology Press; 2014.137–156 p.

[26] Moustakas CE (1961). Loneliness. Englewood Cliffs.

[27] Heinrich LM, Gullone E (2006). The clinical significance of loneliness: a literature review. Clin Psychol Rev.

[28] Lasgaard M. Loneliness in adolescence: conceptualization, methodology, and empirical evidence. Aarhus University; 2010.

[29] Hojat M, Lyon K (1998). Psychosocial characteristics of female students in the Allied Health and Medical Colleges: Psychometrics of the Measures and personality profiles. Adv Heal Sci Educ.

[30] Baumeister RF, Nathan Dewall C, Ciarocco NJ, Twenge JM (2005). Social exclusion impairs self-regulation. J Pers Soc Psychol.

[31] Wilson C, Moulton B. Loneliness among older adults: A national survey of adults 45+. Washington, DC; 2010.

[32] Cornwell EY, Waite LJ (2009). Social disconnectedness, perceived isolation, and health among older adults. J Health Soc Behav.

[33] Rico-Uribe LA, Caballero FF, Martín-María N, Cabello M, Ayuso-Mateos JL, Miret M. Association of loneliness with all-cause mortality: A meta-analysis. Jacobs JM, editor.PLoS One. 2018 Jan4;13(1):e0190033. 10.1371/journal.pone.0190033 PMC5754055 29300743

[34] Yoo-Jeong M, Hepburn K, Holstad M, Haardörfer R, Waldrop-Valverde D. Correlates of loneliness in older persons living with HIV. AIDS Care. 2020 Jul;2(7):869–76. 10.1080/09540121.2019.1659919 PMC7047591 31462066

[35] Harris M, Brouillette MJ, Scott SC, Smaill F, Smith G, Thomas R (2020). Impact of loneliness on Brain Health and Quality of Life among adults living with HIV in Canada. J Acquir Immune Defic Syndr.

[36] van den Deckx L, Buntinx F (2014). Risk factors for loneliness in patients with cancer: a systematic literature review and meta-analysis. Eur J Oncol Nurs.

[37] Savikko N, Routasalo P, Tilvis RS, Strandberg TE, Pitkälä KH. Predictors and subjective causes of loneliness in an aged population. Arch Gerontol Geriatr. 2005 Nov;41(3):223–33. 10.1016/j.archger.2005.03.002 15908025

[38] Brennan J, Gingell P, Brant H, Hollingworth W. Refinement of the Distress Management Problem List as the basis for a holistic therapeutic conversation among UK patients with cancer. Psychooncology. 2012 Dec;21(12):1346–56. 10.1002/pon.2045 21905157

[39] Wells M, Kelly D. The loneliness of cancer.Eur J Oncol Nurs. 2008Dec;12(5):410–1. 10.1016/j.ejon.2008.11.003 19056062

[40] Opoku Agyemang S, Ninonni J, Bennin L, Agyare E, Gyimah L, Senya K et al. Prevalence and associations of depression, anxiety, and stress among people living with HIV: A hospital-based analytical cross‐sectional study. Heal Sci Reports. 2022 Sep 8;5(5). 10.1002/hsr2.754 PMC9358537 35949667

[41] Wright K, Naar-King S, Lam P, Templin T, Frey M. Stigma scale revised: reliability and validity of a brief measure of Stigma for HIV + Youth. J Adolesc Heal. 2007 Jan;40(1):96–8. 10.1016/j.jadohealth.2006.08.001 PMC2001277 17185215

[42] Reinius M, Wettergren L, Wiklander M, Svedhem V, Ekström AM, Eriksson LE (2017). Development of a 12-item short version of the HIV stigma scale. Health Qual Life Outcomes.

[43] Russel W (1996). UCLA LoneThiraess Scale (Version 3): Validity, and factor structure. J Pers Assess.

[44] Russell D, Peplau LA, Ferguson ML (1978). Developing a measure of loneliness. J Pers Assess.

[45] Lee EE, Depp C, Palmer BW, Glorioso D, Daly R, Liu J (2019). High prevalence and adverse health effects of loneliness in community-dwelling adults across the lifespan: role of wisdom as a protective factor. Int Psychogeriatr.

[46] Braun V, Clarke V (2014). What can “thematic analysis” offer health and wellbeing researchers?. Int J Qual Stud Health Well-being.

[47] Lincoln YS, Guba EG, Pilotta JJ. Naturalistic inquiry.Int J Intercult Relations. 1985Jan;9(4):438–9.

[48] Kalomo EN, Jun JS, Lee K, Kaddu MN (2020). HIV stigma, resilience and depressive symptoms among older adults living with HIV in rural Namibia. Afr J AIDS Res.

[49] Beres LK, Narasimhan M, Robinson J, Welbourn A, Kennedy CE. Non-specialist psychosocial support interventions for women living with HIV: A systematic review.AIDS Care. 2017 Sep2;29(9):1079–87. 10.1080/09540121.2017.1317324 PMC6957075 28438030

[50] Kuteesa MO, Wright S, Seeley J, Mugisha J, Kinyanda E, Kakembo F, et al. Experiences of HIV-related stigma among HIV-positive older persons in Uganda – a mixed methods analysis. SAHARA-J J Soc Asp HIV/AIDS. 2014 Jan;2(1):126–37. 10.1080/17290376.2014.938103 PMC4272102 25053275

[51] Rokach A (2014). Loneliness of the marginalized. Open J Depress.

[52] Stutterheim SE, Bos AER, Shiripinda I, de Bruin M, Pryor JB, Schaalma HP (2012). HIV-related stigma in African and Afro-Caribbean communities in the Netherlands: manifestations, consequences and coping. Psychol Heal.

[53] Flickinger TE, DeBolt C, Xie A, Kosmacki A, Grabowski M, Waldman AL (2018). Addressing Stigma through a virtual community for people living with HIV: a mixed methods study of the PositiveLinks Mobile Health intervention. AIDS Behav.

[54] Judgeo N, Moalusi KP. My secret: the social meaning of HIV/AIDS stigma. SAHARA-J J Soc Asp HIV/AIDS. 2014 Jan;2(1):76–83. 10.1080/17290376.2014.932302 PMC4272192 24980478

[55] Jin H, Earnshaw VA, Wickersham JA, Kamarulzaman A, Desai MM, John J et al. An assessment of health-care students’ attitudes toward patients with or at high risk for HIV: implications for education and cultural competency.AIDS Care. 2014 Oct 3;26(10):1223–8. 10.1080/09540121.2014.894616 PMC4089975 24625279

[56] Houtsonen J, Kylma J, Korhonen T, Valimaki M, Suominen T (2014). University Students’ perception of people living with HIV/AIDS: discomfort, fear, knowledge and a willingness to Care. Coll Stud J.

[57] Horter S, Bernays S, Thabede Z, Dlamini V, Kerschberger B, Pasipamire M et al. “I don’t want them to know”: how stigma creates dilemmas for engagement with Treat-all HIV care for people living with HIV in Eswatini.African J AIDS Res. 2019 Jan2;18(1):27–37. 10.2989/16085906.2018.1552163 30782082

[58] Bozkurt O, Bayırlı Turan D (2020). Evaluation of the knowledge and stigmatization level of HIV/AIDS and related factors. J Psychiatr Nurs.

[59] Hatzenbuehler ML, Phelan JC, Link BG (2013). Stigma as a fundamental cause of population health inequalities. Am J Public Health.

[60] Fekete EM, Williams SL, Skinta MD (2018). Internalised HIV-stigma, loneliness, depressive symptoms and sleep quality in people living with HIV. Psychol Heal.

[61] Ware NC, Wyatt MA, Tugenberg T. Social relationships, stigma and adherence to antiretroviral therapy for HIV/AIDS.AIDS Care. 2006 Nov18;18(8):904–10. 10.1080/09540120500330554 17012079

[62] Goffman E (1968). Stigma: notes on the management of spoiled identity.

[63] Ali K, Farrer L, Gulliver A, Griffiths KM. Online peer-to-peer support for Young People with Mental Health problems: a systematic review. JMIR Ment Heal. 2015 May;19(2):e19. 10.2196/mental.4418 PMC4607385 26543923

[64] Yeshua-Katz D. Online Stigma Resistance in the Pro-Ana Community.Qual Health Res. 2015 Oct9;25(10):1347–58. 10.1177/1049732315570123 25667161

[65] Chow EPF, Lau JTF, Zhuang X, Zhang X, Wang Y, Zhang L (2014). HIV Prevalence Trends, Risky Behaviours, and Governmental and Community responses to the epidemic among men who have sex with men in China. Biomed Res Int.

[66] Makoae LN, Greeff M, Phetlhu RD, Uys LR, Naidoo JR, Kohi TW, et al. Coping with HIV-Related Stigma in five african countries. J Assoc Nurses AIDS Care. 2008 Mar;19(2):137–46. 10.1016/j.jana.2007.11.004 PMC2346777 18328964

[67] Makin JD, Forsyth BWC, Visser MJ, Sikkema KJ, Neufeld S, Jeffery B (2008). Factors affecting disclosure in south african HIV-positive pregnant women. AIDS Patient Care STDS.

[68] Brandão BMG, de Angelim M, de Marques RC, de Oliveira SC. Abrão FM da S. Living with HIV: coping strategies of seropositive older adults.Rev da Esc Enferm da USP. 2020;54. 10.1590/s1980-220x2018027603576 32667387

[69] Mattes D. Caught in Transition: The Struggle to Live a ‘Normal’ Life with HIV in Tanzania.Med Anthropol. 2014 Jul4;33(4):270–87. 10.1080/01459740.2013.877899 24422743

